# Can Beach Cleans Do More Than Clean-Up Litter? Comparing Beach Cleans to Other Coastal Activities

**DOI:** 10.1177/0013916516649412

**Published:** 2016-05-19

**Authors:** Kayleigh J. Wyles, Sabine Pahl, Matthew Holland, Richard C. Thompson

**Affiliations:** 1 School of Psychology, Plymouth University, Plymouth, UK; 2 Plymouth Marine Laboratory, Plymouth, UK; 3 School of Marine Science and Engineering, Plymouth University, Plymouth, UK

**Keywords:** marine debris, ocean citizenship, well-being, knowledge, pro-environmental behavior, attention restoration theory

## Abstract

Coastal visits not only provide psychological benefits but can also contribute to the accumulation of rubbish. Volunteer beach cleans help address this issue, but may only have limited, local impact. Consequently, it is important to study any broader benefits associated with beach cleans. This article examines the well-being and educational value of beach cleans, as well as their impacts on individuals’ behavioral intentions. We conducted an experimental study that allocated students (*n* = 90) to a beach cleaning, rock pooling, or walking activity. All three coastal activities were associated with positive mood and pro-environmental intentions. Beach cleaning and rock pooling were associated with higher marine awareness. The unique impacts of beach cleaning were that they were rated as most meaningful but linked to lower restorativeness ratings of the environment compared with the other activities. This research highlights the interplay between environment and activities, raising questions for future research on the complexities of person-environment interactions.

## Introduction

The marine environment, covering over 70% of the Earth’s surface, is exposed to numerous anthropogenic threats. One such global, persistent, and increasing threat is marine litter, manufactured solid waste material that enters the marine environment (Galgani et al., 2010). Marine litter can have lethal and sub-lethal effects on wildlife through entanglement, physical damage, and also potential chemical contamination by ingesting those materials (Browne, Niven, Galloway, Rowland, & Thompson, 2013; Gall & Thompson, 2015; Rochman, Hoh, Kurobe, & Teh, 2013). It can also have negative impacts on visitors, as a common form of visual pollution, potentially undermining visitors’ physical and psychological well-being gained from the coast (Tudor & Williams, 2008). For example, Wyles, Pahl, Thomas, and Thompson (2016) compared littered and clean coastal scenes and found that rubbish potentially left by the public (e.g., food wrappers) was especially disliked, associated with making people feel sad, and diminished the restorative qualities of the environment, as compared with other environmental conditions. As well as being seen as one of the most harmful acts visitors can have on the environment in a recent survey (Wyles, Pahl, & Thompson, 2014), public-litter (rubbish accidentally or deliberately left on the coast or carried there by winds and rivers) is the largest contributor to marine litter (Marine Conservation Society [MCS], 2012).

Recreational visits can be partly responsible for adding to marine litter, but these visits can also have numerous benefits. These benefits include improving well-being, raising awareness about the environment in terms of its ecology and anthropogenic threats, and strengthening an environmental citizenship in the coastal visitors to engage in more pro-environmental behaviors. Spending time by the sea has been found to be restorative, improving individuals’ health and well-being (Ashbullby, Pahl, Webley, & White, 2013; White, Pahl, Ashbullby, Herbert, & Depledge, 2013). These restorative effects can be explained by the Attention Restoration Theory (ART; Kaplan, 1995) that describes why certain settings can psychologically revitalize people (e.g., increasing cognitive attention and well-being). Namely, environments are proposed to be restorative if they give a sense of *being away*, facilitate *fascination*, are rich in *extent*, and are *compatible* with a person’s intention. Coastal environments have been rated highly in terms of these properties (e.g., Hipp & Ogunseitan, 2011; White et al., 2010). These experiences can also be linked to pro-environmental behaviors, as individuals experiencing restorative environments have reported engaging in more ecological behaviors than those who have not experienced these environments (Hartig, Kaiser, & Bowler, 2001; Hartig, Kaiser, & Strumse, 2007). Furthermore, correlational work in the United States by Steel, Lovrich, Lach, and Fomenko (2005) found that individuals who visit this environment have greater awareness about the environment and the stressors facing it. Thus, visits to the coast in general can be seen to have numerous benefits, but do these benefits vary by the specific activities people engage in? Specifically, what is the effect of common leisure activities compared with increasingly popular citizen science activities that include a learning element? Finally, does dealing with a potentially unsavory threat in the environment (such as marine litter) undermine restorativeness of an environment, and/or does it have complementary benefits to the individuals?

Beach cleaning campaigns are arranged around the world, involving individuals volunteering their time and effort to help collect and dispose of the rubbish found on the shore. This marine stewardship activity is sometimes undertaken with the sole aim of improving the condition of the beach, but it can also be part of a wider initiative that involves a citizen science element where data on litter are also systematically recorded (Measham & Barnett, 2008). While these campaigns help to improve the local environment and remove items already in the environment, their contribution to the wider problem may be relatively small (in terms of reducing quantities of marine litter). Consequently, it is necessary to understand their wider benefits, both to the volunteers and in turn to the environment (e.g., well-being and educational value and further benefits for the environment by either encouraging individuals to repeat engagement and/or to perform other pro-environmental acts). To explore these broader benefits of participating in beach cleans, we report a study that compared beach cleans with other coastal activities using a controlled experimental design.

### Literature Review

While there is little research on the broader benefits of beach cleans specifically, studies have examined other stewardship activities and volunteering in general. These studies have looked at both why individuals volunteer and their self-reported outcomes. For example, within the motivational functionalism literature, improving personal well-being and raising awareness are popular motives for volunteering in general (e.g., volunteers want to feel good about themselves, do something meaningful, and expand their knowledge and understanding; Bramston, Pretty, & Zammit, 2011; Clary et al., 1998; Evans et al., 2005; Grese, Kaplan, Ryan, & Buxton, 2000; Katz, 1960; R. L. Ryan, Kaplan, & Grese, 2001). If these motivations are satisfied, the individuals are then more likely to volunteer again in the future (Asah & Blahna, 2012; Clary et al., 1998). Thus, according to research on volunteers’ motivations, well-being and learning are factors central to volunteering, with other studies beginning to examine these specific outcomes but rarely examining them together.

Well-being outcomes have been reported in a range of volunteering programs. These studies indicate that volunteers experience greater hedonic well-being benefits (relating to pleasure), such as greater positive emotion, compared with those who do not volunteer (Borgonovi, 2008; Meier & Stutzer, 2008; Piliavin & Siegl, 2007; Thoits & Hewitt, 2001). While these pleasure-focused benefits are insightful, it is important to also stress the eudaimonic well-being benefits (the level of meaningfulness). R. M. Ryan and Deci (2001) have argued that both hedonic and eudaimonic factors enhance well-being. In relation to a broader range of activities including paid work, child care, and so on, volunteering was found to be the most meaningful and rewarding activity (White & Dolan, 2009). Studies examining general volunteering have found that these activities correlate more strongly with eudaimonic well-being than hedonic (Son & Wilson, 2012). When examining volunteers of a marine monitoring program, which involved gathering data about marine biota in Australia, volunteers reported experiencing both hedonic and eudaimonic well-being benefits after the experience (Koss & Kingsley, 2010). For example, when evaluating the program, volunteers typically noted that they enjoyed the work; felt emotionally good, calm, and peaceful; and found that it gave them meaning (Koss & Kingsley, 2010). Thus, previous literature has shown that environmental stewardship activities and volunteering in general are associated with hedonic and, especially, eudaimonic well-being. However, the causal direction of these effects is currently unclear, and it is unclear whether this also applies to activities in the marine environment such as beach cleans in particular that include dealing with items of rubbish.

As well as potential well-being benefits, beach cleans may also increase individuals’ knowledge or awareness about marine litter (Bravo et al., 2009; Grese et al., 2000; Kordella, Geraga, Papatheodorou, Fakiris, & Mitropoulou, 2013; R. L. Ryan et al., 2001). Many of the beach cleaning organizations have the goal or assume that volunteers will leave such events with greater awareness about this environmental issue (Bravo et al., 2009; Kordella et al., 2013). Theoretically, this is a plausible assumption as learning about a topic in the appropriate context has been found to be more effective than learning in more abstract environments such as classrooms (Duerden & Witt, 2010); however, few studies explicitly measure this. Evans and colleagues (2005) did a basic evaluation of a bird recording program on volunteers. They found that volunteers reported an increased level of knowledge regarding bird biology and overall environmental awareness after engaging in the program. In this and other broader studies examining environmental awareness, measures are primarily subjective, involving individuals judging their own levels of knowledge (e.g., Steel et al., 2005) or retrospectively self-assessing if they felt they have learnt something (e.g., Clary et al., 1998). To date, there has been no research examining the impact beach cleans can have on subjective awareness nor on the impacts of environmental stewardship activities more broadly on objective marine awareness (actual knowledge and understanding of the marine environment).

An ultimate goal of beach cleans (and other marine stewardship activities) is to encourage a continued commitment to that activity (Cunningham & Snowden, 2008; Hidalgo-Ruz & Thiel, 2013; Uneputty, Evans, & Suyoso, 1998). For instance, one study monitored volunteers’ behavior after a beach cleaning event in Indonesia and found that volunteers continued to pick up rubbish and not drop rubbish themselves a couple of months later (Uneputty et al., 1998). Self-report surveys have also found that current volunteers often intend to volunteer again in the future (Cunningham & Snowden, 2008; Hidalgo-Ruz & Thiel, 2013). As well as encouraging a continued commitment to the volunteering program, other more generic pro-environmental acts can also be encouraged. For example, engaging in one stewardship act may encourage individuals to adopt other environmental citizenship behaviors, such as more sustainable energy use (e.g., Thøgersen & Ölander, 2003). This would therefore illustrate a potential positive spillover effect, which is an indirect side effect of an intervention, behavior, or process (Poortinga, Whitmarsh, & Suffolk, 2013; Thøgersen & Ölander, 2003). Thus, participating in beach cleans may result in greater commitment to these programs and may even trigger more pro-environmental behaviors in general.

In sum, beach cleans may provide broader benefits beyond improving the condition of the local environment (e.g., increasing well-being and marine awareness for the individuals engaging in the act and encouraging future pro-environmental behaviors—both directly by intending to take part in future beach cleans and in more generic pro-environmental behaviors implying a positive spillover). However, previous methods adopted to examine these relationships heavily depend on correlational approaches, and they rarely examine beach cleaning specifically. For example, these studies commonly collect the outcome measures outside of the activity context and correlate with whether individuals have volunteered in the past year or the number of hours volunteered (Borgonovi, 2008; Grese et al., 2000; Koss & Kingsley, 2010; Meier & Stutzer, 2008; Piliavin & Siegl, 2007; R. L. Ryan et al., 2001; Son & Wilson, 2012; Thoits & Hewitt, 2001). Consequently, the direct impacts of engaging in these specific stewardship activities are not explicitly examined and may suffer from selection effects by using existing volunteers. Pretest–posttest designs would be appropriate to deduce a more direct relationship, by measuring variables immediately before and after engaging in the activity, ideally with novices to reduce expectation effects.

To be able to establish whether there are any unique impacts of beach cleans, it is also necessary to compare beach cleans with other activities. For example, beach cleans may be found to be beneficial simply because they involve spending time in an environment associated with these benefits (Ashbullby et al., 2013; Hartig et al., 2001; Hartig et al., 2007; Steel et al., 2005; White et al., 2013) rather than for the activity itself, as currently argued in the well-being literature. Activities in general can be seen to differ in both hedonic and eudaimonic well-being (White & Dolan, 2009), while in the coastal setting, some studies find that certain activities were perceived to bring greater well-being benefits (Wyles et al., 2014) with others implying that all leisure activities are similarly beneficial if they take place in the same environmental context (White et al., 2013). Thus, to further explore the unique benefits of beach cleans to the individuals and the environment, it would be better to directly compare beach cleans with other coastal activities.

### Present Research

To be able to infer the unique impacts of beach cleans, we used a student sample with little to no beach cleaning experience, who were allocated to one of three carefully selected activities: beach cleaning, rock pooling, or coastal walking. The rock pooling activity, the exploration of pools of water on the shore, involved both an exploration and a citizen science element to it, not only making it similar to that of the beach cleaning activity but also representing a popular activity undertaken at the coast. As an activity for comparison, walking was selected as it is the most popular activity undertaken in natural environments in England (Natural England, 2010). By assigning participants to one of three activities (they were initially only informed that they would be participating in an unspecified coastal activity), the study applied an experimental approach to address the more direct effects associated with beach cleans, and monitored well-being, marine awareness, and behavioral intention immediately before and after the activity, and again a week later.

This article consequently aims to address one overarching research question:

**Research Question 1:** What are the broader benefits of participating in beach cleans other than to remove rubbish from the shore?

Specifically, we explored five sub-questions: first, what is the well-being impact of beach cleans (examining change in both hedonic and eudaimonic well-being and the perceived restorative quality of the environment)? Second, what is the educational impact of beach cleans, in terms of raising (a) general subjective marine awareness and (b) objective awareness about marine litter specifically (by measuring a change in awareness about marine litter but also a comparative subject: marine biodiversity). Third, is there a change in intention to volunteer in beach cleans in the future and to engage in generic environmental behaviors? Fourth, how do beach cleans compare to other coastal activities? And finally, do any effects remain one week after the event?

## Method

### Site

This experimental field study took place at Mount Batten Bay, in the south of Devon, United Kingdom, less than 5 km (3 miles) from Plymouth (see Online Appendix A). The upper shore is predominantly sand and shingle, with the mid shore consisting of solid rock. During low tide, the beach is approximately 170 m wide. It is accessible to visitors, with easy access from roads and public transport; has a coastal path run parallel to the shoreline; and has facilities such as parking, toilets, and food nearby.

### Participants

Participants were recruited using a university psychology undergraduate points system in exchange for course credits. Participants needed to be physically fit and mobile, have normal or corrected vision, and have suitable walking shoes and weather appropriate clothing. Ninety-two participants were recruited (22 male, 69 female, one non-reported), but due to withdrawals (one participant for ill-health and another because of objecting to the beach clean activity), the final sample consisted of 90 participants (21 male, 68 female, one non-reported) with an average age of 22 years (*SD* = 6.18). Many (46%) participants reported visiting this particular type of coastline once or twice a year, with the majority of participants walking (82%), relaxing (64%), and socializing (63%) during those visits. Thirty participants were in each of the three activity groups, and demographics and baseline measures were similar across the three groups (*p*s > .13, see Table 1 for more information).

**Table 1.** Demographic Information for Each Activity (*N* = 90).

	Beach cleaning (*n* = 30)	Rock pooling (*n* = 30)	Coastal walking (*n* = 30)
Gender	8 male, 22 female	5 male, 24 female, 1 non-reported	8 male, 22 female
Age	21.30 (*SD* = 3.54)	20.38 (*SD* = 3.36)	23.23 (*SD* = 9.38)
Frequency of rocky shore visits	47% = once or twice a year	40% = once or twice a year	50% = once or twice a year
Most common activities performed when on shore	Walking Relaxing Socializing	Walking Socializing Relaxing	Walking Relaxing Socializing/Eating
Experience in these activities^a^	0% Beach cleaning 57% Rock pooling 87% Coastal walking	3% Beach cleaning 40% Rock pooling 87% Coastal walking	0% Beach cleaning 23% Rock pooling 73% Coastal walking

a Percentage of participants who reported doing those activities.

### Design

**Figure 1.**
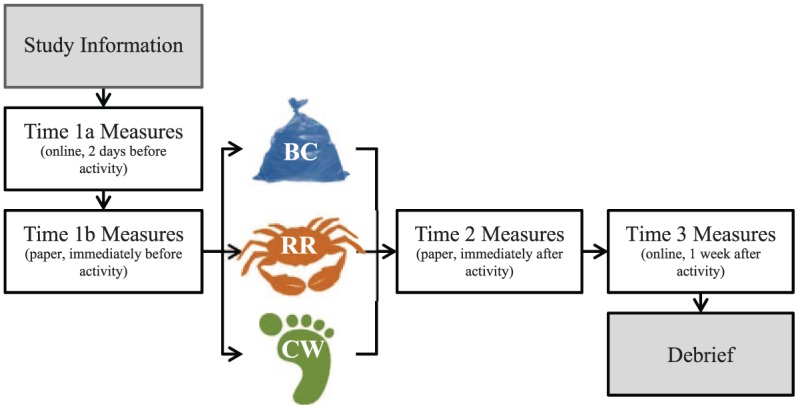
A schematic diagram of the methodological design, with participants completing measures before, immediately after, and a week after engaging in one of three activities: BC, RP, or CW. *Note.* BC = beach cleaning; RP = rock pooling; CW = coastal walking.

Measures were obtained at three time points: before the activity (Time 1, T1a and T1b), immediately after (Time 2, T2), and one week later (Time 3, T3; Figure 1), and were completed individually. Activities lasted approximately 90 minutes in total, were performed as groups (ranging from two to 12 participants), and kept independent to the research surveys. To reduce any selection and expectation effects, participants signed up for a study on coastal activities. They were only told the specific activity they were assigned to after the baseline measures had been obtained. Due to constraints regarding tidal conditions, weather, and expert availability, we alternated activities by day (beach cleaning, rock pooling, coastal walking). The activities occurred over 10 days between September and November 2012 on days when low tide fell between 10:00 and 13:00 so that the visible intertidal area and daylight levels were standardized.

### The Activities

#### Beach cleaning

This activity replicated the MCS beach cleaning citizen science program. Permission and resources were given by the MCS (L. Davis, personal communication, September 11, 2012). A marine biologist ran this activity to keep the psychological surveys independent from the activity. Each session began with a standardized briefing lasting 15 minutes, where the marine biologist summarized the issue of marine litter (e.g., time it takes to degrade and common sources) and introduced the citizen science program by describing the program, its relevance, where the data go, and how it is used to tackle marine litter. Participants were then briefed on how to record the litter. The equipment was distributed among the participants, who were encouraged to work in pairs or threes. Participants were then free to record and collect marine litter, with help from the marine biologist. After an hour, the participants were gathered to tally up the results and discuss what they found as a group. Marine litter data collected were sent to the MCS to contribute to their national dataset.

#### Rock pooling

To be comparative to the beach cleaning activity, this activity also involved a citizen science component along with representing a popular activity undertaken at the coast. Consequently, also led by the marine biologist, rock pooling involved a briefing and debriefing either side of (a) a free-style rock pooling session and (b) a citizen science session. The marine biologist first explained the importance of the current habitat and offered some facts regarding the organisms that live there. The citizen science aspect was then introduced, using the Shore Thing program. Shore Thing is a nationwide survey and involves a timed species search where abundance levels for 32 specific species are recorded within a 20 minute period (Marine Biological Association, 2012). After explaining the best practice in the environment, participants were free to explore the rocky shore in small groups (pairs or threes) for 30 minutes (a). Afterward, for the Shore Thing species survey (b), each participant was allocated two to three species to search for in a particular area of the shore, with the aid of the marine biologist and descriptive identification cards. At the end of this timed search, participants were required to report back to the marine biologist the overall level of abundance for each of their allocated species. To conclude the activity, the marine biologist debriefed everyone, summarizing the most common species found. The data were then sent to the national Shore Thing database for analysis to monitor the nationwide impact of rising sea temperature on coastal species.

#### Coastal walking

This activity was different to the former two, as it did not have a citizen science aspect and did not require such a standardized briefing, debriefing, or the expertise of a qualified marine biologist. Consequently, this activity was led by one of two data collectors, who explained to the group that they would be walking along the coastal path. Participants were fore-warned of the reasonably steep, potentially slippery terrain, and were consequently advised to walk at a comfortable pace, taking breaks where needed. Walking in small groups, participants walked 35 minutes along the coastal path (see Online Appendix A) before walking back. Typically, walks involved four short breaks and covered just over 3.5 km (2.17 miles).

### Experimental Materials

Baseline measures were collected using online surveys for the more stable constructs (e.g., marine awareness, behavioral intentions, and demographics) two days before (Time 1a) and paper surveys for the more temporally sensitive measures (e.g., mood) immediately before the activity (Time 1b). Well-being measures were recorded again at T2 along with perceived restorativeness; with marine awareness and behavioral intentions measured again at T2 and T3. The specific items are described below. Other measures (connectedness to nature, leisure time, relationship with fellow participants) were collected but were not of key interest for the current article, thus will not be addressed.

#### Well-being and restorativeness

To examine well-being effects, three measures were used. Hedonic well-being was measured with a mood scale at T1b and T2 (e.g., White & Dolan, 2009). Participants rated how strongly they felt positive (happy, content/relaxed, calm) and negative (nervous/anxious, sad/depressed, frustrated) emotions on a scale from *not at all* (1) to *extremely* (7; T1b positive affect Cronbach’s α = .84, negative affect α = .51; and T2 positive affect α = .85, negative affect α = .60).^1^ An overall mood variable was then calculated based on the affect-balance tradition (Bradburn, 1969) by subtracting negative affect from positive affect, resulting in scores ranging from −6 to +6.

To examine participants’ overall evaluation of the activity at T2 and T3, a single-item satisfaction measure asked *all things considered, how satisfied are you with today’s [last week’s] activity* ranging from *very unsatisfied* (1) to *very satisfied* (10; adapted from White & Dolan, 2009).

Eudaimonic well-being (meaningfulness) was also measured after the event at T2 and T3 by participants rating their level of agreement to whether the activity was *worthwhile and meaningful to me* and *in line with my values* (similar to that in White & Dolan, 2009) on a 7-point rating scale from *not at all* (1) to *extremely* (7; Spearman–Brown coefficients at T2 = .79 and T3 = .84).

In addition to measuring well-being directly, it was of interest whether the perceived restorativeness of the environment differed across the activities. A short modified version of the Perceived Restorativeness Scale (Hartig, Korpela, Evans, & Gärling, 1997) was used based on ART (Kaplan, 1995). As used in White and colleagues (2010), immediately after the activity, participants rated their level of agreement to four statements: (a) *This site is a place which is away from everyday demands and where I would be able to relax and think about what interests me* (being away); (b) *This place is fascinating; it is large enough for me to discover and be curious about things* (fascination); (c) *This site is a place which is very large, with no restrictions to movements; it is a world of its own* (extent); (d) *Here, it is easy to orient and move around so that I could do what I like* (compatibility), on a scale from *completely disagree* (1) to *completely agree* (7), Cronbach’s α = .83.

#### Marine awareness

Three forms of marine awareness were used: first, general subjective marine awareness was examined; second, objective marine awareness regarding marine litter was assessed; and finally, as the rock pooling activity also involved an educational citizen science component and to examine whether changes in marine awareness is specific to the topic of marine litter, a supplementary objective marine awareness on intertidal biodiversity was also examined. Subjective awareness involved participants rating their level of awareness about (a) *overall biology (the science of life) of the shore*, (b) *natural threats faced by organisms (such as damage from storms) on the shore*, (c) *general human-induced challenges facing shore organisms (e.g., oil spills)*, and (d) *the specific visitor-induced threats to shore organisms (e.g., from walking)* on a scale from *not at all informed* (1) to *high expertise* (5; as used in Wyles et al., 2014). When combined to form an overall scale of subjective marine awareness, this was highly reliable at each of the three time points (Cronbach’s α at T1a = .82, T2 = .87, T3 = .85).

Five multiple choice questions were constructed to assess different aspects of objective awareness of marine litter (based on the publicly accessible literature; MCS, 2011; a similar approach to Steel et al., 2005). This included questions on the most common type and source of litter found, the amount found annually, and the time it takes for items to biodegrade, which were not found to be too easy or difficult when initially piloted (see Online Appendix B for individual items and their scores). Percentage of correct responses was then calculated to produce an overall correct percentage score for each person at each time point.

A similar approach was used for objective marine awareness in relation to biodiversity. This included a multiple choice item regarding the definition of ecology and correctly identifying species as being native to the United Kingdom. For the latter aspect, participants were shown nine pictures of intertidal species and had to identify whether those species can be found in the U.K. waters. All were native to the United Kingdom but were systematically chosen so that species varied in colorfulness, difficulty, and likelihood to be found if rock pooling (as previously piloted; see Online Appendix C). Similar to above, percentages of correct responses were then calculated for each participant at each time point.

#### Behavioral intentions

Behavioral intentions were measured on a scale from *never* (1) to *all of the time* (5) in response to the question *in the future, how often do you think you will engage in the following behaviors?* In addition to single items examining participants’ intentions to engage in beach cleans, rock pooling, and coastal walks in the future, a generic 11 item pro-environmental behavior scale was used. The scale comprised of items that varied in difficulty and type of behavior, such as *persuade friends to lead a more sustainable lifestyle; when walking in nature, I will take care where I tread*; and *support sustainable policies with petitions and political vote* (Boomsma, 2013; Stern, 2000). This scale resulted in a highly reliable measure of overall pro-environmental intention at each of the three time points (Cronbach’s α for T1a = .80, T2 = .85, T3 = .87).

### Procedure

Participants were emailed the online T1a survey two days before the visit to the coast. This included the detailed brief explaining that they would complete surveys before and after a structured trip to the coast lasting around two hours. On the day of the activity, participants met at the university, where additional written consent and health and safety information were collected. As a group, they were then led on foot and via a short water taxi ride to the site. Once at the site, they completed the well-being T1b measures and were then informed which activity they would complete. Participants completed that day’s activity, followed by the T2 survey. Participants were emailed the T3 survey seven days later.

### Analysis

Analysis first involved screening the data (e.g., checking for normality and statistical outliers). As some data were non-normally distributed, both non-parametric and parametric tests were used in all analyses, with the latter reported unless conclusions differed. After screening the data, preliminary checks were performed. There were no main effects of day of activity, age, or gender on any of the main variables (*p*s > .08), apart for subjective marine awareness whereby self-reported awareness was negatively correlated with age, *F*(1, 85) = 6.80, *p* = .01, $\eta_{p}^{2}$ = .07 (small effect^2^).

To examine both changes over time and differences between activities, mixed ANOVAs were applied, with time (T1, T2, and T3) as a within-subject variable, and activity (beach cleaning, rock pooling, coastal walking) as the between-subject variable for each of the key outcomes. To explore statistically significant main effects further, Sidak post hoc tests were used to explore main effects of time (when sphericity was not violated, and Bonferroni within-subjects post hoc tests when it was), and Bonferroni post hoc tests for the main effects of activity (all of which report values after adjusting for familywise error). Simple effects analyses were conducted for significant interactions, involving breaking the interaction down and running tests on each component (e.g., three sets of one-way ANOVAs looking at the variable over the three time points for each of the activities). To manually control for familywise error for these simple effects analyses, a more stringent *p* value was adopted (typically dividing the standard *p* value of .05 by three, the number of analyses ran).

## Results

### Well-Being and Restorativeness

The visit to the coast, regardless of activity, was rated highly for all measures of well-being (Table 2). Mood was positive at T1b and T2 and did not change over time (*p* = .94). Mood ratings were also similar across the activities (*p* = .44), and there was no interaction between activity and time (*p* = .07). Overall satisfaction was also rated positively after the event but participants’ ratings statistically declined somewhat a week later, *F*(1, 86) = 11.30, *p* = .001, $\eta_{p}^{2}$ = .12 (small effect). Satisfaction ratings did not differ between activities (*p* = .45) nor was the interaction between time and activity significant (*p* = .33).The eudaimonic well-being measure, meaningfulness, did not change from T2 to T3 (*p* = .33), and the interaction was not statistically significant (*p* = .15), but there were statistical differences between the three activities, *F*(2, 86) = 5.11, *p* = .008, $\eta_{p}^{2}$ = .11 (small effect). Bonferroni post hoc tests identified one statistical difference between conditions: that beach cleaning was perceived as more meaningful than walking (*p* = .006).

**Table 2.** The Means (and *SD*) for Well-Being and Marine Awareness Measures for Each Activity (*n* = 30) Over Three Time Periods.

Condition	Time point		
	Time 1	Time 2	Time 3
Well-being: Mood—*very negative* (−6) to *very positive* (+6)			
Beach cleaning	3.25 (1.77)	2.67 (1.95)	—
Rock pooling	3.40 (1.85)	3.24 (1.97)	—
Coastal walking	3.18 (1.86)	3.82 (1.64)	—
Total	3.28 (1.81)	3.24 (1.90)	—
Well-being: Satisfaction—*very unsatisfied* (1) to *very satisfied* (10)			
Beach cleaning	—	7.20 (1.71)	6.48 (2.49)
Rock pooling	—	7.70 (1.86)	7.30 (1.84)
Coastal walking	—	7.30 (2.04)	7.00 (1.84)
Total	—	7.40 (1.87)^a^	6.93 (2.08)^b^
Well-being: Meaningfulness—*not at all* (1) to *extremely* (7)			
Beach cleaning^1^	—	5.33 (1.14)	5.09 (1.21)
Rock pooling^1,2^	—	4.68 (1.28)	4.77 (1.13)
Coastal walking^2^	—	4.37 (1.07)	4.35 (1.15)
Total	—	4.79 (1.22)	4.73 (1.19)
Well-being: Perceived restorativeness—*completely disagree* (1) to *completely agree* (7)			
Beach cleaning^1^	—	4.19 (1.35)	—
Rock pooling^1,2^	—	4.66 (1.27)	—
Coastal walking^2^	—	5.03 (0.89)	—
Total	—	4.63 (1.22)	—
Marine awareness: Subjective marine—*not at all informed* (1) to *high expertise* (5)			
Beach cleaning	2.36 (0.71)^a^	2.86 (0.88)^b^	2.72 (0.77)^b^
Rock pooling	2.61 (0.52)^a^	2.96 (0.68)^b^	3.02 (0.66)^b^
Coastal walking	2.47 (0.74)	2.54 (0.70)	2.59 (0.61)
Total	2.48 (0.66)^a^	2.79 (0.77)^b^	2.78 (0.70)^b^
Marine awareness: Objective litter marine (% correct)			
Beach cleaning	52.67 (20.67)	50.00 (21.50)	51.33 (21.45)
Rock pooling	48.00 (20.07)	44.67 (23.89)	48.00 (18.64)
Coastal walking	44.67 (26.09)	45.33 (26.23)	54.67 (25.69)
Total	48.44 (22.43)	46.67 (23.80)	51.33 (22.04)
Marine awareness: Objective biodiversity marine (% correct)			
Beach cleaning^1^	54.24 (10.26)	54.33 (13.57)	60.61 (12.25)
Rock pooling^2^	60.61 (16.94)	65.00 (20.47)	69.39 (16.62)
Coastal walking^1^	54.24 (13.40)	56.00 (14.99)	55.45 (12.71)
Total	56.36 (13.98)^a^	58.44 (17.09)^a,b^	61.82 (15.00)^b^

*Note*. Superscripts are used to illustrate the post hoc analyses (significant differences are illustrated by having no superscript in common): superscripts a and b are used to illustrate within-subject post hoc analysis for exploring the main effect of time or the interaction between time and activity, whereas 1, 2, and 3 are used to illustrate between-subject analysis comparing conditions.

As well as reporting positive experiences as a result of this visit to the coast, participants perceived this environment to have a high restorative value (Table 2). However, these perceptions differed depending on the activity participants engaged in, *F*(2, 87) = 3.79, *p* = .03, $\eta_{p}^{2}$ = .08 (small effect). Upon further investigation, Bonferroni post hoc analysis found participants in the beach cleaning group rated the environment less restorative than those engaging in the coastal walk (*p* = .02; see Table 2).

### Marine Awareness

At T1a, participants felt they knew the basics regarding marine topics (see Table 2). For subjective marine awareness, ratings increased from T1a to T2 and remained high one week later (T3), *F*(2, 174) = 16.80, *p* < .001, $\eta_{p}^{2}$ = .16 (small effect). Specifically, subjective marine awareness was rated higher at T2 and T3 than at T1a (*p*s < .001; see Table 2). There was no significant main effect of activity (*p* = .12); however, a statistically significant interaction arose, *F*(4, 174) = 2.51, *p* = .04, $\eta_{p}^{2}$ = .05 (small effect). Simple effects analysis using a more stringent *p* value of *p* = .017 to adjust for familywise error found that there was only a main effect of time for beach cleaning, *F*(2, 58) = 10.04, *p* < .001, $\eta_{p}^{2}$ = .26 (medium effect), and rock pooling, *F*(1.35, 39.26) = 9.31, *p* = .006, $\eta_{p}^{2}$ = .24 (medium effect), while ratings did not change for the coastal walking group (*p* = .42, see Table 2). For both beach cleaning and rock pooling, subjective marine awareness significantly increased from T1a to T2 (*p*s < .04), and remained at this higher level at T3 (*p*s > .58).

To see whether these findings were also reflected in the objective measures of marine awareness, participants’ accuracy on the multiple choice questions were examined. As shown in Table 2, no significant patterns emerged from the data for the litter-based questions. The main effect of time and activity, and the interaction effect were all not statistically significant (*p*s > .19).

To see whether participants’ marine awareness changed for another topic (especially relevant for the rock pooling activity), the multiple choice questions on biodiversity were explored. In contrast to the litter-related awareness questions, significant differences arose for these biodiversity questions. First, the main effect for time was found to be significant, *F*(2, 174) = 5.85, *p* = .003, $\eta_{p}^{2}$ = .06 (small effect). Correct responses increased over time, but statistically, only responses at T3 had significantly improved from T1a (*p* = .001; see Table 2). There was also a main effect for activity, *F*(2, 87) = 5.97, *p* = .004, $\eta_{p}^{2}$ = .12 (small effect), whereby rock pooling participants were more accurate in their responses overall than both the beach cleaning (*p* = .02) and the walking groups (*p* = .007). However, the interaction between time and activity was not found to be statistically significant (*p* = .12).

### Behavioral Intentions

For behavioral intentions (to engage in the three activity-specific behaviors in the future and to carry-out pro-environmental behaviors in general), the same pattern emerged over time: intentions increased from T1a to T2 and declined slightly at T3 a week later (see Table 3). We found a statically significant change over time for all intentions: to engage in beach cleans, *F*(1.81, 155.85) = 17.84, *p* < .001, $\eta_{p}^{2}$ = .17 (using Huynh–Feldt as sphericity was violated); rock pooling, *F*(2, 172) = 6.262, *p* = .002, $\eta_{p}^{2}$ = .07 (small effect); and coastal walks, *F*(1.95, 165.62) = 9.40, *p* < .001, $\eta_{p}^{2}$ = .10 (small effect, using Huynh–Feldt), as well as for the more general pro-environmental behaviors, *F*(1.85, 159.46) = 61.26, *p* < .001, $\eta_{p}^{2}$ = .42 (large effect, using Huynh–Feldt). Post hoc analyses found that all intentions increased significantly from T1a to T2 (*p*s < .004). These intentions remained higher at T3 than at T1a, with the exception of the intention to go rock pooling, which dropped to a similar level as T1a (*p* < .03, see Table 3). While intentions to engage in generic pro-environmental behaviors did decline slightly from T2 to T3 (*p* = .002), participants’ intentions to engage in the activity-specific behaviors (beach cleaning, rock pooling, and coastal walking) remained high at T3 (statistically similar to T2; *p*s > .17).

**Table 3.** The Means (and *SD*) for Behavioral Intention Measures—Range: *Never* (1) To *All of the Time* (5)—for Each Activity (*n* = 30) Over Three Time Periods.

Condition	Time point		
	Time 1	Time 2	Time 3
Intention to engage in a *beach clean* in the future			
Beach cleaning	2.07 (0.78)	2.87 (0.73)^1^	2.55 (1.02)
Rock pooling	1.93 (0.69)	2.23 (0.90)^2^	2.23 (0.82)
Coastal walking	2.03 (0.72)	2.23 (0.68)^2^	2.23 (0.63)
Total	2.01 (0.73)^a^	2.44 (0.82)^b^	2.34 (0.84)^b^
Intention to go *rock pooling* in the future			
Beach cleaning	3.30 (0.84)	3.47 (0.90)	3.24 (0.83)
Rock pooling	3.10 (0.96)	3.50 (1.01)	3.33 (1.03)
Coastal walking	2.77 (0.97)	3.23 (0.86)	3.10 (0.84)
Total	3.06 (0.94)^a^	3.40 (0.92)^b^	3.22 (0.90)^a,b^
Intention to go *coastal walking* in the future			
Beach cleaning	3.57 (1.19)	3.73 (0.87)	3.69 (0.89)
Rock pooling	3.43 (1.17)	3.90 (0.92)	3.63 (1.03)
Coastal walking	3.23 (0.86)	3.62 (0.98)	3.60 (1.04)
Total	3.41 (1.08)^a^	3.75 (0.92)^b^	3.64 (0.98)^b^
Intention to engage in *environmentally responsible* behaviors in the future			
Beach cleaning	2.82 (0.50)	3.34 (0.49)	3.19 (0.55)
Rock pooling	2.81 (0.62)	3.25 (0.68)	3.13 (0.75)
Coastal walking	2.78 (0.52)	3.07 (0.60)	2.99 (0.60
Total	2.80 (0.54)^a^	3.22 (0.60)^b^	3.10 (0.64)^c^

*Note*. Superscripts are used to illustrate the post hoc analyses (significant differences are illustrated by having no superscript in common): superscripts a, b, and c are used to illustrate within-subject post hoc analysis for exploring the main effect of time, whereas 1 and 2 are used to illustrate between-subject analysis comparing conditions during the post hoc or simple effects analyses.

All intentions were similar across activities (*p*s > .07), and, apart from intentions to engage in beach cleans, there were no significant interactions (*p*s > .11). For the intention to engage in a beach clean in the future, a significant interaction arose, *F*(3.62, 155.85) = 3.40, *p* = .01, $\eta_{p}^{2}$ = .07 (small effect, using Huynh–Feldt). Simple effects analysis found that all groups responded similarly at T1a and T3 (*p*s > .25), but differed at T2 (*p* = .01). Specifically, participants had a greater intention to engage in a beach clean in the future after engaging in that particular activity compared with participants who did the rock pooling or coastal walking activities (*p*s < .01).

In summary, this study used an experimental design to investigate the impact of beach cleans compared with two other coastal activities. Participants did not know beforehand which activity they were going to engage in. Mood and satisfaction were rated highly for all activities, but satisfaction declined significantly a week later at T3. Meaningfulness was also rated highly, remained high a week later, and was found to be more pronounced for the beach cleaning activity. The environment was rated positively in terms of restorative qualities, but with ratings lowest for the beach cleaning activity. For marine awareness, subjective awareness increased after the visit to the coast, more so for the beach cleaning and rock pooling groups; however, when examining objective awareness, an increase was only found for the biodiversity-related objective measures. Positively, intention to engage in generic pro-environmental acts was found to increase for all activities but the effects may be relatively short-term, as intentions started to drop a week later. In addition, the intention to engage in beach cleans in the future were higher for those who participated in that activity, but this differentiation between groups disappeared a week later, implying a relatively short-lived heightened intention.

## General Discussion

Marine litter is a prominent global issue that can be harmful to the environment and also to coastal visitors. A direct solution for local areas is beach cleaning campaigns where volunteers help to collect and dispose of the rubbish found. However, these campaigns will have a relatively small contribution to addressing this issue as a whole; therefore, it is necessary to understand beach cleaning initiatives’ wider benefits. Understanding the impacts on individuals’ well-being, marine awareness, and even behavioral intentions is important individually and also collectively to build a more holistic picture of these broader benefits. Consequently, this article examined the benefits of participating in a beach clean in terms of changes in well-being, marine awareness, and intentions to volunteer in future beach cleans and other pro-environmental behaviors over time and compared with other coastal activities.

Using an experimental design, we found positive outcomes for hedonic and eudaimonic well-being, irrespective of the activity. While individuals were satisfied with the visit, arrived happy, and left similarly happy, similar to Koss and Kingsley’s (2010) study, the current findings do not reflect previous findings where mood improves for leisure activities more generally (e.g., Ashbullby et al., 2013; White et al., 2013). This might be because the benefits from the environment and/or the *anticipation* of spending time on the coast were already apparent at baseline. These findings could also relate to the *dose-response effect*, where our participants may not have experienced the optimum amount of time for an individual to receive the most benefit from an environment (e.g., White et al., 2010; White et al., 2013). When compared with coastal walking and rock pooling, beach cleaning was only found to be different for the eudaimonic well-being measure (higher) and perceived restorativeness (lower). The lack of activity differences in hedonic well-being could support the argument that it is the coastal environment rather than the activity that is beneficial for (hedonic) well-being (White et al., 2013). In contrast, eudaimonic well-being was more pronounced for the beach cleaning activity, in line with the previous correlational research on volunteering in general (Son & Wilson, 2012; White & Dolan, 2009). Thus, this article demonstrates a unique meaningfulness benefit among coastal activities which was associated with beach cleans specifically.

Beach cleans were also found to be different from the other activities in terms of perceived restorativeness, but in a more negative way. Compared with participants engaging in a coastal walk who rated the environment positively in terms of ART’s properties (Kaplan, 1995), individuals participating in the beach clean rated the environment as more neutral. Three explanations could apply. First, the restorativeness may have been constrained by the work-like nature of the beach cleaning activity. While ART is proposed to be applicable to all individuals (Kaplan, 1995), von Lindern, Bauer, Frick, Hunziker, and Hartig (2013) found that forest workers did not benefit to the same extent as non-forest workers when visiting forests for leisure. However, von Lindern et al. (2013) specifically found that the perceived restorativeness was reduced mainly due to the lower ratings of *being away* which had been compromised as the sites were more familiar to the forest workers. Consequently, this is different from a volunteer context, especially with our student sample who had not participated in a beach clean in the past and have only previously undertaken leisure activities at this type of coastline. Second, even though these activities were both undertaken in a similar overall context (the coast), the activities focused on different aspects of the environment (e.g., the coastal walk covered more ground and types of habitats thus highlighting the breadth and extent of this environment, whereas the beach clean focused on a specific area of shore, and on litter specifically). Finally, this lower perceived restorativeness rating could be related to previous research that found litter can degrade the restorative quality of an environment (Pretty, Peacock, Sellens, & Griffin, 2005; Wyles et al., 2016). Thus, engaging in an act that emphasizes this non-pristine feature would be expected to result in lower restorativeness ratings. However, if a (salient) littered environment was to have such powerful impacts on people’s well-being as suggested by previous laboratory studies (Pretty et al., 2005; Wyles et al., 2015), it is conceivable that participants engaging in this activity would also have poorer well-being as a result of the activity. Yet, hedonic well-being was found to be the same as for the other activities, and eudaimonic well-being was found to be better. This could suggest either that litter does detract from the restorative properties of a coast but is not as detrimental to well-being in situ, or alternatively, that the *environment* itself is less restorative but the *activity* is beneficial to well-being, thus counteracting the potential harmful impacts. However, these explanations are purely speculative. Future research is suggested to further look at activity differences (to date, no other study has examined how the perceived restorativeness ratings vary depending on activity) along with exploring its relation with well-being.

Subjective marine awareness was also seen to improve after visiting the coast. This not only supports previous literature that visiting the coast increases marine awareness (Settar & Turner, 2010; Steel et al., 2005) but also identifies distinctive awareness improvements for specific activities. In comparison with walking, we found similar results for beach cleaning and rock pooling. As both the activities with a citizen science element found an increase, this supports the general goal of citizen science to engage and educate (Bravo et al., 2009; Eastman, Núñez, Crettier, & Thiel, 2013; Kordella et al., 2013) and extends similar findings from other programs (e.g., Evans et al., 2005). Even better, this higher subjective awareness was also found to remain a week later.

The findings for the subjective measure of marine awareness are partially mirrored by the objective measures, however, only for the biodiversity-related questions for the rock pooling group. This can extend previous studies that completing this type of citizen science program (identifying marine biota) does not just improve people’s subjective awareness but also their more objective awareness (Evans et al., 2005; Koss & Kingsley, 2010). The explanation behind the lack of significant effects for the marine litter–related questions is less clear. It could be inferred that the procedures linked with this event were simply not as effective as those for the rock pooling group.

Intentions to engage in generic pro-environmental behaviors were seen to increase, declining slightly one week on from the coastal visit, but still remaining higher than baseline. Similar to Koss and Kingsley (2010), participants of the two citizen science activities left with greater intention to change their behavior to protect the natural environment; however, this was also the case for the coastal walking group. As all three activity groups responded similarly, this impact appears not to be activity dependent. This could indicate that it is the environment that is influential in these outcomes rather than the activity, as viewing waterscape images can have similar outcomes (Hartig et al., 2001). Overall, this is an encouraging finding that engaging with the environment, at any level, can potentially empower people’s intention and desire to act more responsibly towards the environment. Those who engaged in a beach clean also expressed a greater intention to volunteer in future beach cleans compared with the two other groups. This is in line with past beach cleaning events, where volunteers express an intention to continue volunteering (Cunningham & Snowden, 2008; Hidalgo-Ruz & Thiel, 2013; Uneputty et al., 1998). Unfortunately, this potential effect appears to only be temporary, as all three groups were similar a week later. Consequently, this identifies that an optimum time to encourage volunteers to commit to a future event is immediately after a beach clean.

### Limitations, Future Work, and Implications

Future research may wish to develop the methods and/or extend the sample. For example, the objective marine awareness measures that were tailored to the current research design (focusing on marine litter and biodiversity) may contribute to the mixed findings reported above. For instance, the biodiversity measure predominantly focused on identifying species, whereas the marine litter measures took a broader approach examining people’s objective awareness about different elements of the issue. Thus, it could be suggested that the differences in the results could be a consequence of measuring different types of awareness. Future work developing these measures could help to disentangle these possible interpretations. Future research should also examine actual behavior change. We would hope that these reported intentions would translate into actual behaviors, but even though intentions are often correlated with behaviors (e.g., Ajzen, 1985; Bamberg & Schmidt, 2003), actual behavior was not directly examined in this article.

The sample was carefully selected for the purpose of this research; however, different populations could be investigated to generalize these findings further. For example, current volunteers could have been recruited to improve the ecological validity; however, this would have provided additional biases, such as recruitment biases and expectation effects, and, as a more heterogeneous sample, would have introduced other potential confounds (such as experience and motives for volunteering). Consequently, we adopted an experimental design using a non-beach cleaning volunteer sample of students and compared three activities using a between-subject design. This student sample was convenient, easily accessible, and meant socio-demographic influences were minimized. Future research could apply experimental methods to a broader, ideally representative general public sample.

Finally, these results may be useful for beach cleaning organizers to help increase repeated commitment and encourage new volunteers. As outlined by the motivational functionalism literature, individuals are more likely to volunteer again in the future if their motives have been successfully met such as feeling good about oneself or learning something new (Asah & Blahna, 2012; Clary et al., 1998). Although we did not measure motivations due to focusing on a non-volunteer sample, we have shown that individuals find the beach cleaning experience meaningful and worthwhile, and leave with greater (subjective) awareness and, to some extent, intention to engage in future events. These insights could be used to help promote these events to recruit new volunteers, emphasizing that the volunteers also receive personal benefits. One method to address people who have not volunteered before could be via initiative schemes or community engagement strategies in businesses to encourage people to initially try it. For example, a supermarket company has previously partnered up with MCS in the United Kingdom to encourage more people to volunteer, by incentivizing volunteers with vouchers and drinks and snacks during the event. This particular campaign resulted in more than 9,000 volunteers in 2013 (M&S, 2014). As demonstrated by our results on pro-environmental intentions, it would then be optimal to invite volunteers to commit to a future beach clean immediately after the initial event, as intentions weakened a week later.

## Conclusion

The marine environment is facing numerous anthropogenic stressors, including marine litter. This article focused on a specific pro-environmental activity that addresses marine litter: beach cleaning. While the immediate benefit on the local environment is evident, we systematically explored the potential broader benefits of this act by comparing beach cleaning with other coastal activities. Overall, it was found that individuals were satisfied with their experience, found it meaningful, felt they had learned more about the marine environment, and intended to engage in more pro-environmental behaviors; however, some of the impacts were similar for rock pooling and coastal walking groups. The unique impacts attributed to beach cleans were that the environment was perceived as less restorative when engaging in a beach clean, but the activity was found more meaningful than the others. Beach cleaning individuals’ subjective marine awareness also increased more than that of participants who went on a coastal walk (but was similar to the other activity with a citizen science element). Thus, there is some evidence not only that the specific activity plays a role but also that spending time in this coastal environment more generally is associated with benefits (e.g., experiencing positive mood and high pro-environmental intentions). In sum, beach cleans designed to tackle this environmental issue are not only beneficial for the local coastline and its habitants. They appear to have a wider educational value that may bring further environmental benefits in the future. In addition, they were shown to benefit individual well-being and strengthen individuals’ environmental citizenship by increasing pro-environmental behavioral intention, at least in the short-term. This research provides further evidence for the complex interplay between environment and person and suggests that the effects of different activities in the natural environment (and their links to well-being and awareness) merit further research.

## Supplementary Material

Supplementary material

## Notes

1. While positive affect was found to be highly reliable, the negative affect items were below the typical acceptable values of alpha (α > .70; Field, 2005). Consequently, we additionally analyzed positive affect and negative affect scores independently. As the conclusions were similar, the overall affect-balance scores are reported for conciseness and in line with Bradburn (1969).

2. According to Cohen’s (1992) criteria, $\eta_{p}^{2}$ value of .10 is a small effect, medium is .25, and large effects have a value of .40 or higher.
