# Supplementary material for: Can Beach Cleans Do More Than Clean-Up Litter? Comparing Beach Cleans to Other Coastal Activities
Source: Environ Behav. 2016 May 19;49(5):509–35. doi: 10.1177/0013916516649412 (PMC5431367; doi:10.1177/0013916516649412)
Supplement: Supplementary material [file supplementary_appendices.docx]

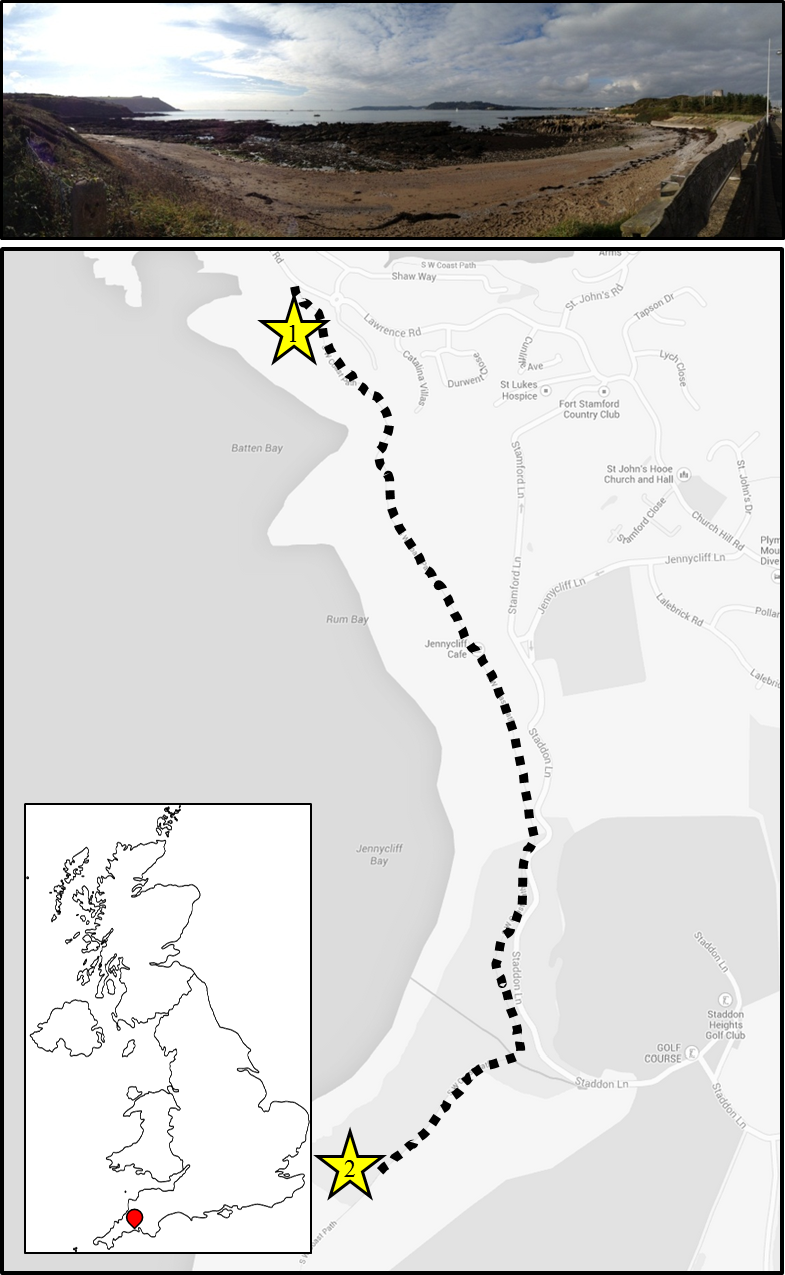


*Appendix A.* The site used, located in the south west of England. All activities began at the entrance of the beach, illustrated in the photograph and located at point 1 on the map, with the beach clean and rock pool groups continuing down to the shore. The walking group, in contrast, walked along the coastal path, as shown with the dotted line in the map (background map sourced from Google Maps, 2013; photograph used with permission of the author, all rights reserved).

Appendix B. *The Frequency (and Percentage) of Correct Responses for Each of the Multiple-Choice Questions Examining Objective Marine Awareness on Marine Litter (n = 90).*

| **Question** | **Condition** | **Baseline** | | **After** | | **Follow-up** | |
| --- | --- | --- | --- | --- | --- | --- | --- |
| Q: What do you think was the most common type of litter found on the UK coastline in 2011?  A: Public litter (left by the public on the coast or inland, which is carried by winds and rivers) | BC | 24 | (80%) | 21 | (70%) | 22 | (73%) |
|  | RR | 27 | (90%) | 24 | (80%) | 27 | (90%) |
|  | CW | 22 | (73%) | 24 | (80%) | 26 | (87%) |
|  | **Total** | **73** | **(81%)** | **69** | **(77%)** | **75** | **(83%)** |
| Q: Regarding individual items, what do you think were the most common items found on the UK coastline in 2011?  A: Plastic pieces | BC | 14 | (47%) | 12 | (40%) | 15 | (50%) |
|  | RR | 7 | (23%) | 9 | (30%) | 10 | (33%) |
|  | CW | 13 | (43%) | 11 | (37%) | 17 | (57%) |
|  | **Total** | **34** | **(38%)** | **32** | **(36%)** | **42** | **(47%)** |
| Q: Over the last 10 years, plastic bottles found on UK beaches have…  A: Increased by 33% | BC | 15 | (50%) | 17 | (47%) | 14 | (47%) |
|  | RR | 12 | (40%) | 8 | (27%) | 8 | (27%) |
|  | CW | 14 | (47%) | 15 | (50%) | 14 | (47%) |
|  | **Total** | **41** | **(46%)** | **40** | **(44%)** | **36** | **(40%)** |
| Q: On average in 2011, how many pieces of litter were found per kilometre?  A: 1,741 pieces | BC | 14 | (47%) | 14 | (47%) | 15 | (50%) |
|  | RR | 15 | (50%) | 11 | (37%) | 13 | (43%) |
|  | CW | 7 | (23%) | 9 | (30%) | 12 | (40%) |
|  | **Total** | **36** | **(40%)** | **34** | **(38%)** | **40** | **(44%)** |
| Q: How long do you think a disposable nappy (diaper) takes to decompose?  A: 75-450 years | BC | 12 | (40%) | 11 | (37%) | 11 | (37%) |
|  | RR | 11 | (37%) | 15 | (50%) | 14 | (17%) |
|  | CW | 11 | (37%) | 9 | (30%) | 13 | (43%) |
|  | **Total** | **34** | **(38%)** | **35** | **(39%)** | **38** | **(42%)** |

Appendix C. *The Frequency (and Percentage) of Correct Responses for Each of the Multiple-Choice Questions Examining Objective Marine Awareness on Biodiversity (n = 90).*

| **Question** | **Condition** | **Baseline** | | **After** | | **Follow-up** | | **Question** | **Condition** | **Baseline** | | **After** | | **Follow-up** | |
| --- | --- | --- | --- | --- | --- | --- | --- | --- | --- | --- | --- | --- | --- | --- | --- |
| Q: The scientific study of interactions among organisms and between organisms and their environment is….?  A: Ecology | BC | 22 | (73%) | 24 | (80%) | 27 | (90%) | Painted top shell | BC | 10 | (33%) | 12 | (40%) | 16 | (53%) |
|  | RR | 26 | (87%) | 22 | (73%) | 24 | (80%) |  | RR | 13 | (43%) | 21 | (70%) | 28 | (93%) |
|  | CW | 25 | (83%) | 27 | (90%) | 25 | (83%) |  | CW | 8 | (27%) | 10 | (33%) | 10 | (33%) |
|  | **Total** | **73** | **(81%)** | **73** | **(81%)** | **76** | **(84%)** |  | **Total** | **31** | **(34%)** | **43** | **(48%)** | **54** | **(60%)** |
| Common prawn | BC | 26 | (87%) | 25 | (83%) | 27 | (90%) | Purple sea urchin | BC | 19 | (63%) | 11 | (37%) | 17 | (57%) |
|  | RR | 28 | (93%) | 24 | (80%) | 27 | (90%) |  | RR | 16 | (53%) | 18 | (60%) | 21 | (70%) |
|  | CW | 25 | (83%) | 25 | (83%) | 27 | (90%) |  | CW | 13 | (43%) | 12 | (40%) | 11 | (37%) |
|  | **Total** | **79** | **(88%)** | **74** | **(82%)** | **81** | **(90%)** |  | **Total** | **48** | **(53%)** | **41** | **(46%)** | **49** | **(54%)** |
| Cushion star fish | BC | 20 | (67%) | 15 | (50%) | 19 | (63%) | Rock goby | BC | 26 | (87%) | 19 | (63%) | 27 | (90%) |
|  | RR | 20 | (67%) | 18 | (60%) | 24 | (80%) |  | RR | 25 | (83%) | 19 | (63%) | 18 | (60%) |
|  | CW | 19 | (63%) | 20 | (67%) | 19 | (63%) |  | CW | 25 | (83%) | 23 | (77%) | 23 | (77%) |
|  | **Total** | **59** | **(66%)** | **53** | **(59%)** | **62** | **(69%)** |  | **Total** | **76** | **(84%)** | **61** | **(68%)** | **68** | **(76%)** |
| Dog whelk | BC | 30 | (100%) | 29 | (97%) | 30 | (100%) | Sea scorpion | BC | 9 | (30%) | 10 | (33%) | 12 | (40%) |
|  | RR | 28 | (93%) | 29 | (97%) | 30 | (100%) |  | RR | 12 | (40%) | 8 | (27%) | 11 | (37%) |
|  | CW | 28 | (93%) | 23 | (77%) | 28 | (93%) |  | CW | 7 | (23%) | 6 | (20%) | 12 | (40%) |
|  | **Total** | **86** | **(96%)** | **81** | **(90%)** | **88** | **(98%)** |  | **Total** | **28** | **(31%)** | **24** | **(27%)** | **35** | **(39%)** |
| Green sea urchin | BC | 13 | (43%) | 12 | (40%) | 13 | (43%) | Snakelocks anemone | BC | 4 | (13%) | 6 | (20%) | 12 | (40%) |
|  | RR | 20 | (67%) | 14 | (47%) | 22 | (73%) |  | RR | 12 | (40%) | 22 | (73%) | 24 | (80%) |
|  | CW | 17 | (57%) | 13 | (43%) | 16 | (53%) |  | CW | 12 | (40%) | 9 | (30%) | 12 | (40%) |
|  | **Total** | **50** | **(56%)** | **39** | **(43%)** | **51** | **(57%)** |  | **Total** | **28** | **(31%)** | **37** | **(41%)** | **48** | **(53%)** |
